# ANCA-Negative Granulomatosis with Polyangiitis Presenting with Hypertrophic Cranial Pachymeningitis, Abducens Nerve Palsy, and Stenosis of the Internal Carotid Artery

**DOI:** 10.1155/2017/9687383

**Published:** 2017-11-30

**Authors:** Shohei Harabuchi, Nobuyuki Bandoh, Rika Yasukawa, Michihisa Kono, Takashi Goto, Yasuaki Harabuchi, Hidetoshi Ikeda, Hajime Kamada, Hiroshi Nishihara

**Affiliations:** ^1^Department of Otolaryngology-Head and Neck Surgery, Hokuto Hospital, Inadacho Kisen 7-5, Obihiro 080-0833, Japan; ^2^Department of Otolaryngology-Head and Neck Surgery, Asahikawa Medical University, Midorigaoka-Higashi 2-1-1-1, Asahikawa 078-8510, Japan; ^3^Department of Neurosurgery, Hokuto Hospital, Inadacho Kisen 7-5, Obihiro 080-0833, Japan; ^4^Department of Biology and Genetics, Laboratory of Cancer Medical Science, Hokuto Hospital, Inadacho Kisen 7-5, Obihiro 080-0833, Japan

## Abstract

We report a rare case of granulomatosis with polyangiitis (GPA) presenting with hypertrophic cranial pachymeningitis (HCP), abducens nerve palsy, and stenosis of the internal carotid artery (ICA). A 59-year-old Japanese man presented with a year history of nasal obstruction and a 2-month history of slight headache. Histopathological examination of the granulomatous mucosa in the ethmoid sinuses resected by endoscopic sinus surgery revealed necrotizing vasculitis with multinucleated giant cells. The patient was diagnosed with the limited form of GPA as a result of the systemic examination. He declined immunosuppressive treatment. Eighteen months after the diagnosis of GPA, he presented with diplopia and severe headache. Though nasal findings indicating GPA were not observed in the nasal cavity, CT scan revealed a lesion of the right sphenoid sinus eroding the bone of the clivus. Gadolinium-enhanced MRI of the brain showed thickening of the dura mater around the right cavernous sinus and clivus. Magnetic resonance angiography and cerebral angiography revealed narrowing at the C5 portion of the ICA. Intravenous methylprednisolone pulse therapy followed by oral prednisolone and cyclophosphamide resolved headache and dramatically improved HCP and stenosis of the ICA.

## 1. Introduction

Granulomatosis with polyangiitis (GPA), formerly known as Wegener's granulomatosis, is a systemic autoimmune disease involving small- to medium-sized vasculitis and necrotizing granulomatous inflammation in the upper and lower respiratory tract, as well as the kidney. Central nervous system (CNS) involvement is uncommon, occurring in 4–18% of GPA patients [1, 2], and characterized by hypertrophic cranial pachymeningitis (HCP) [3, 4], cranial nerve paralysis [4], cerebral infarction [5–7], and subarachnoid hemorrhage [8, 9]. Here, we report a rare case of GPA presenting with HCP, abducens nerve palsy, and stenosis of the internal carotid artery (ICA).

## 2. Case Report

**Figure 1:**
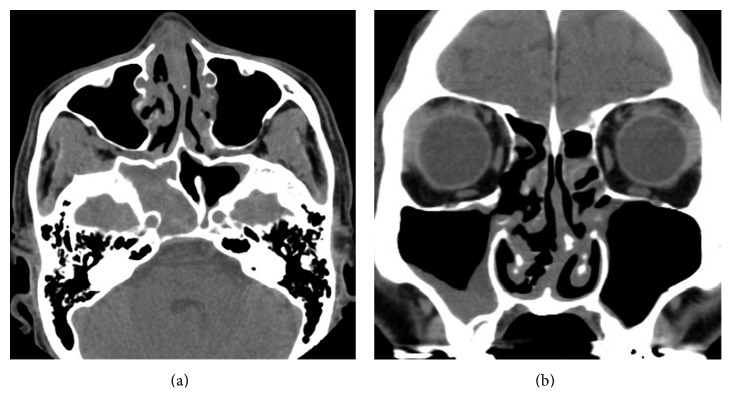
CT scan at first visit shows swelling of the mucosa in the bilateral ethmoid sinuses (a) along with a soft-tissue area occupying the right sphenoid sinus (b).

**Figure 2:**
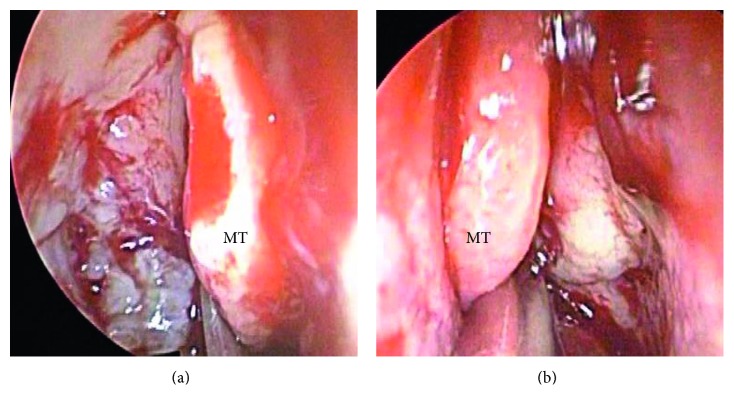
During endoscopic sinus surgery, granulomatous mucosa is apparent in the right (a) and left (b) ethmoid sinuses.

**Figure 3:**
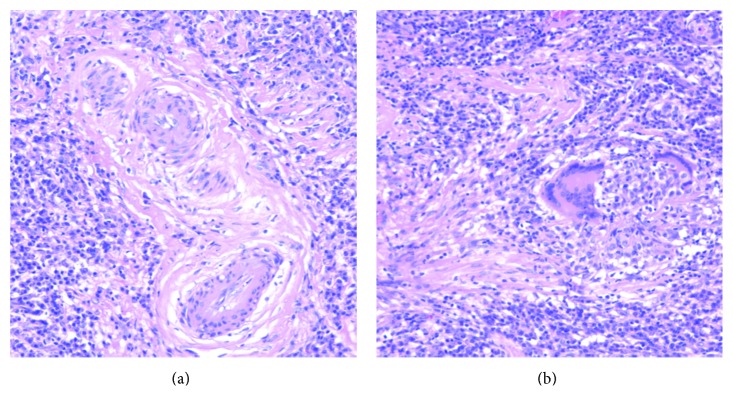
Histopathological examination of the granulomatous mucosa from the ethmoid sinus shows significant necrotizing vasculitis (a) and granulomatous inflammation with multinucleated giant cells (b) (HE staining, ×200). MT: middle turbinate.

A 59-year-old Japanese man presented with a year history of nasal obstruction and a 2-month history of slight headache. Nasal examination showed swelling and crust formation of the bilateral inferior turbinate. CT scan revealed swelling of the mucosa in the bilateral ethmoid sinuses and soft-tissue filling in the right sphenoid sinus (Figures 1(a) and 1(b)). We initially diagnosed chronic rhinosinusitis and performed endoscopic sinus surgery (ESS) under general anesthesia. We opened the bilateral ethmoid and the sphenoid sinuses. During ESS, easy bleeding and granulomatous mucosa in the bilateral ethmoid sinuses was seen and resected (Figures 2(a) and 2(b)). Histopathological examination of the mucosa revealed necrotizing vasculitis with multinucleated giant cells (Figures 3(a) and 3(b)). We then examined the patient systemically. Brain MRI and chest CT scan showed no abnormal finding. Urinalysis revealed neither hematuria nor proteinuria. Bilateral ear drums were normal. No hearing or visual loss was identified. Negative results for serum proteinase 3- (PR3-) and myeloperoxidase- (MPO-) anti-neutrophil cytoplasm antibody (ANCA) were obtained from fluorescence enzyme immunoassay (FEIA). We diagnosed the limited form of GPA. Shortly after making this diagnosis, the headache resolved and nasal obstruction disappeared. The patient declined immunosuppressive treatment for fear of worsening of his diabetes mellitus. We started careful follow-up every 3 months.

**Figure 4:**
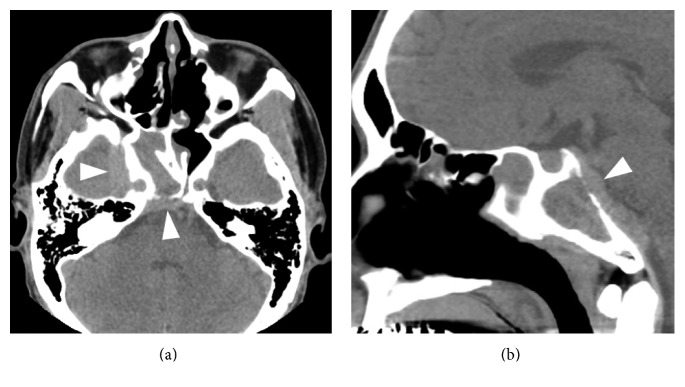
CT scan 18 months after being diagnosed with GPA shows a lesion of the right sphenoid sinus eroding the bone of the clivus and thickening of the dura mater on axial (a) and sagittal (b) images (triangle).

**Figure 5:**
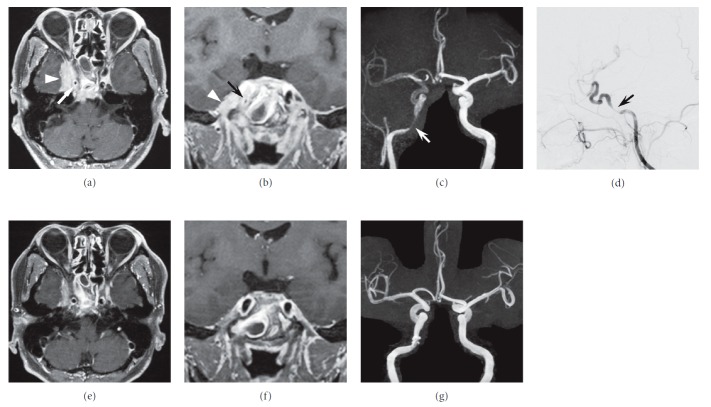
Imaging studies before immunosuppressive treatment (a–d) and 2 years after the initiation of the treatment (e–g). Gadolinium-enhanced T1-weighted MRI of the brain reveals thickening of enhanced dura mater extending from the cavernous sinus to the clivus on axial (a) and coronal (b) images (triangle). Narrowing at the C5 portion of the internal carotid artery (ICA) is apparent on axial (a) and coronal (b) MRI, MRA (c), and lateral cerebral angiography of the ICA (d) (arrow). Thickening of the dura mater and stenosis of the ICA improved on axial (e) and coronal (f) MRI and MRA (g).

Eighteen months after the diagnosis of GPA, the patient presented with diplopia and sudden onset of severe right-sided headache. Nasal endoscopic examination revealed only slight edematous mucosa in the bilateral nasal cavity and the ethmoid sinus. The opening of the right sphenoid sinus was closed by the granulation covered with mucosa, and we could not look into the inside of the sinus. CT scan revealed a lesion of the right sphenoid sinus eroding the bone of the clivus and thickening of the dura mater (Figures 4(a) and 4(b)). Gadolinium-enhanced MRI of the brain showed thickening of the dura mater around the right cavernous sinus and clivus (Figures 5(a) and 5(b)). Magnetic resonance angiography (MRA) and cerebral angiography revealed narrowing at the C5 portion of the ICA (Figures 5(a)–5(d)). Results for serum PR3- and MPO-ANCA were negative. We diagnosed HCP and right abducens nerve palsy, and stenosis of the intracranial ICA due to GPA extension. The patient did not show ischemic attack. Intravenous methylprednisolone (mPSL) pulse therapy was administered at 1g/day for 3 days, followed by oral prednisolone (PSL) at 30 mg/day and cyclophosphamide (CY) at 50 mg/day. The PSL dose was gradually tapered. The headache disappeared immediately. One year after the initiation of the treatment, the patient showed slight saddle nose deformity but did not complain of nasal pain and obstruction. Brain MRI and MRA showed a reduction in the thickness of the dura mater and improved blood flow in the ICA (Figures 5(e)–5(g)). Although the patient still has right abducens nerve palsy, he has not complained of headache for 2 years under a medication with oral PSL at 7 mg/day and azathioprine at 25 mg/day.

## 3. Discussion

This report highlights a unique case of GPA with CNS involvement. First, the patient was diagnosed with not only HCP around the cavernous sinus and right abducens nerve palsy but also stenosis of the intracranial ICA due to extension of GPA from the paranasal sinus. Second, neither serum PR3-ANCA nor MPO-ANCA has been detected at any stage during the course. Third, this patient with HCP, stenosis of the ICA, and headache was successfully treated with immunosuppressive treatment without causing ischemic attack or cerebral infarction. HCP, which occurs in 0.6–8% of GPA patients [1, 2], is characterized by localized or diffuse thickening of the intracranial dura mater [4]. Gadolinium-enhanced MRI is the most useful diagnostic technique for the detection of HCP. HCP is most commonly seen in the falx cerebri and tentorium cerebelli and is rare around the cavernous sinus [1]. Cranial nerve paralysis occurs in about 50% of GPA patients with HCP. The II, III, V, VI, VII, and X cranial nerves are predominantly affected [4, 10]. To the best of our knowledge, only 12 cases with GPA accompanied by cerebrovascular disorder, including the present case, have been reported in the literature as summarized in Table 1 [3, 5–9, 11–13]. Headache was the most common symptom and reported in 7 (58%) of 12 cases. HCP and nose, ear, eye, renal, and lung involvement were shown in 7 (58%) and 7 (58%), 5 (42%), 7 (58%), 6 (50%), and 4 (33%) of 12 cases, respectively. Only 2 cases showed intracranial ICA stenosis accompanied by HCP around the cavernous sinus and cranial nerve paralysis like the present case [11, 12].

CNS involvement in GPA is explained by any combination of 3 possible mechanisms: (i) direct extension from nasal and paranasal sinuses to adjacent structures; (ii) vasculitis of the CNS; and (iii) formation of remote granulomatous lesions in the CNS [14]. In the present case, we suspected that both direct extension of inflammation and vasculitis spread out from the ethmoid and sphenoid sinuses to the cavernous sinus, dura mater, and abducens nerve. Narrowing of the ICA was supposed to be caused by luminal stenosis by vasculitis as well as compression of the surrounding thickened dura mater.

GPA is usually associated with serum PR3- or MPO-ANCA, with the sensitivity and specificity of more than 95% in patients with active generalized GPA [15]. ANCA titer offers an indicator of disease activity or relapse after treatment [4]. There have been a certain number of case reports regarding GPA patients who were negative for both MPO- and PR3-ANCAs. The 2012 Chapel Hill consensus conference definitions proposed ANCA-negative ANCA-associated vasculitis caused by three possibilities: (i) ANCA that cannot be detected by current methods; (ii) ANCA of as yet undiscovered specificity; and (iii) pathological mechanisms not involving ANCA [16]. We cannot exclude the third possibility but can nominate the former two possibilities as candidates. A large-scale study has shown that 83% of ANCA-negative GPA patients show severe CNS involvement, whereas only 10% of all the GPA patients have CNS involvement [1], suggesting that GPA patients with serum ANCA negativity are closely associated with CNS involvement [1].

In the present case, the opportunity to identify HCP and stenosis of the ICA was missed for eighteen months, because we had considered the GPA status of the patient to be stable due to the lack of symptoms and of nasal findings indicating GPA at scheduled follow-up and ANCA negativity. Closure of the opening of the right sphenoid sinus may have worsened the granulomatous inflammation in the sinus and delayed the detection of the disease extension by nasal endoscopy. Ischemic attack was able to be prevented in advance by treatment with mPSL pulse therapy followed by oral PSL and CY. Rapid disease remission can be achieved by any of the treatment strategies that include mPSL pulse therapy, PSL, CY, plasma exchange, and rituximab [2, 4]. Patients with GPA need maintenance treatment with PSL and other immunosuppressive agents such as azathioprine and methotrexate to avoid relapses for a long period [14]. GPA usually has a chronic relapsing and remitting course. Careful follow-up with nasal endoscopic examination, CT scan, and MRI is necessary to find out the progression of GPA and any side effects by immunosuppressive agents.

## 4. Conclusion

HCP and cerebrovascular disorder must be taken into consideration when a patient with GPA present with severe headache and cranial nerve paralysis. Gadolinium-enhanced MRI is quite useful for the diagnosis and follow-up of HCP. The combination of corticosteroid and immunosuppressant is required for induction and remission in patients with GPA.

## Figures and Tables

**Table 1:** Characteristics of reported cases with granulomatosis with polyangiitis (GPA) accompanied by cerebrovascular disorder.

Author	Year	Age/sex	Clinical features	Cerebrovascular involvement	HCP	Nose	Ear	Eye	Renal	Lung	MPO-ANCA	PR3-ANCA	Treatment	Outcome
Cruz and Segal [8]	1997	71/M	Headache, nausea, CN VII	SAH	−	−	+	−	+	+	+	nd	mPSL, CY	Improved

Nagashima et al. [11]	2000	53/F	Paraplagia, fever, CN I, II, VI, VII, VIII	ICA stenosis, loss of ophthalmic artery	+	−	+	+	+	−	+	+	PSL, AZP	Dead

Thajeb and Tsai [12]	2001	65/M	Headache, hyperesthesia, fever, CN III, V	ICA stenosis, cavernous sinus syndrome	+	+	+	+	−	−	−	+	PSL, CY	Improved

Sivakumar and Chandrakantan [5]	2002	49/M	Hemiparesis, seizure, fever, CN X	Infarction of pons and temporal lobe	−	+	+	+	+	−	nd	+	CY	Improved

Fam et al. [3]	2003	63/F	Headache, CN II	Narrowing of ophthalmic artery	+	+	−	+	−	−	+	−	PSL, CY, MTX	Improved

Weijtens et al. [13]	2004	45/F	Hearing loss, Horner's syndrome, CN VI, X	Cavernous sinus involvement	+	+	−	+	−	−	+	−	DEX, AZP	Improved

Takei et al. [9]	2004	34/M	Headache, fever	SAH	−	+	−	−	+	+	−	+	mPSL, PSL, CY	Improved

Peng and Wang [6]	2012	58/M	Headache, ataxia, motor weakness, meningeal signs, CN III, VI, IX, X	Cerebral infarction	+	−	−	+	+	+	+	+	mPSL, PSL, CY	Improved

Yajima et al. [7]	2015	75/M	Headache, hemiparesis, loss of consciousness, hoarseness, hearing loss, fever	Hemorrhagic infarction	+	+	+	−	+	+	nd	+	Frontal lobectomy, mPSL, PSL, AZP	Improved

Present case	2017	59/M	Headache, CN VI	ICA stenosis	+	+	−	+	−	−	−	−	mPSL, PSL, CY, AZP	Improved

CN: cranial nerve affected, SAH: subarachnoid hemorrhage, ICA: internal carotid artery, HCP: hypertrophic cranial pachymeningitis, +: affected or positive, −: not affected or negative, nd: not detected, mPSL: methylprednisolone, CY: cyclophosphamide, PSL: prednisolone, AZP: azathioprine, MTX: methotrexate, DEX: dexamethasone.
